# Functional Connectivity of Successful Picture-Naming: Age-Specific Organization and the Effect of Engaging in Stimulating Activities

**DOI:** 10.3389/fnagi.2020.535770

**Published:** 2020-11-05

**Authors:** Perrine Ferré, Julien Jarret, Simona Brambati, Pierre Bellec, Yves Joanette

**Affiliations:** ^1^Centre de Recherche de l’institut de Gériatrie de l’Université de Montréal (CRIUGM), Montréal, QC, Canada; ^2^Département de Psychologie, Université de Montréal, Montréal, QC, Canada

**Keywords:** functional connectivity, picture-naming, aging, cognitive reserve, mediation analysis

## Abstract

Aging is a lifelong process that starts at birth. Throughout the course of their life, individuals are exposed to various levels of stimulating activities. A higher level of engagement in such activities is suspected to protect against the normal course of cognitive aging or the cognitive manifestations of age-related brain diseases. However, the exact mechanism underlying such protective action remains unclear. The concept of the neurocognitive reserve was introduced to refer to the hypothesis that engagement in stimulating activities shapes brain structure and function, thus indirectly allowing for better preserved cognitive abilities. Although it is known that word production is among the best-preserved cognitive abilities in aging, the underlying neurofunctional mechanisms that allow this relative preservation are still unknown, and it is still unclear how engagement in stimulating activities affects these processes. The objective of this study is to describe the brain functional connectivity patterns associated with picture-naming abilities in younger and older adults with varying levels of engagement in stimulating activities, as a proxy for neurocognitive reserve. A mediation analysis was applied to determine whether the association between reserve proxies and naming accuracy is dependent on task FC. Results show that naming accuracy depends on the posterior cingulate cortex (PCC) functional decoupling in both younger and older adults but through different pathways. While high-performing older adults rely on the asynchronization of this area from motor speech regions’ activity, the best-performing younger adults rely on the functional decoupling with language-related regions. Mediation analysis reveals that the PCC decoupling mediates the relationship between the level of engagement in stimulating activities and naming accuracy in younger adults, but not in older adults. These findings suggest that reserve-related mechanisms may be more critical for naming in early adult life, while older adults’ neurofunctional organization may benefit more from a lifetime of acquired knowledge.

## Introduction

The World Health Organization estimates that the number of individuals aged 60 and over will increase to two billion by 2050, which represents twice the current number for the general population (United Nations, 2015). This demographic change represents a challenge regarding the preservation of functional health (WHO, 2018), which is a crucial factor for well-being. However, not all individuals appear to follow the same aging trajectory, with some maintaining high cognitive performance longer than others. In this context, a better understanding of the mechanisms that support cognitive performance in aging, as well as the influence of engagement in stimulating activities, may be key in identifying the optimal conditions to support lifelong cognitive health and in potentially delaying the expression of cognitive impairments in the context of age-related brain diseases.

Interindividual differences in cognitive preservation in older adulthood appear to result from a complex interplay between genetic and environmental factors (Yang et al., 2016). While our understanding of the genetic dimensions of aging continues to progress, the translation of this knowledge into clinical interventions is slow. In the meantime, engagement in stimulating activities and other environmental factors represent promising protective factors, as they are modifiable (e.g., Stern, 2002). Research on environmental factors may therefore open up new avenues for preventive actions that support optimal cognitive aging. Lifelong engagement in a variety of activities such as education, occupational attainment, or leisure activities has been suggested to protect cognitive function in older age (Anthony and Lin, 2018). These activities are also associated with a lower risk of developing mild cognitive impairment (MCI) or dementia (Scarmeas et al., 2003; Valenzuela and Sachdev, 2006; Stern, 2012; Franzmeier et al., 2016).

Nevertheless, it remains unclear exactly how engagement in stimulating activities might support cognition in aging. The most widely held hypothesis is that engaging in stimulating activities indirectly supports cognitive performance by enhancing the capacity and efficiency of neural activity, thus making the brain more resilient to further impairment (Steffener et al., 2012; Cabeza et al., 2018; Stern et al., 2020). This phenomenon is captured under the general term “neurocognitive reserve,” defined as “the accumulation of brain resources during the lifespan” (Cabeza et al., 2018, p. 707).

**Figure 1:**
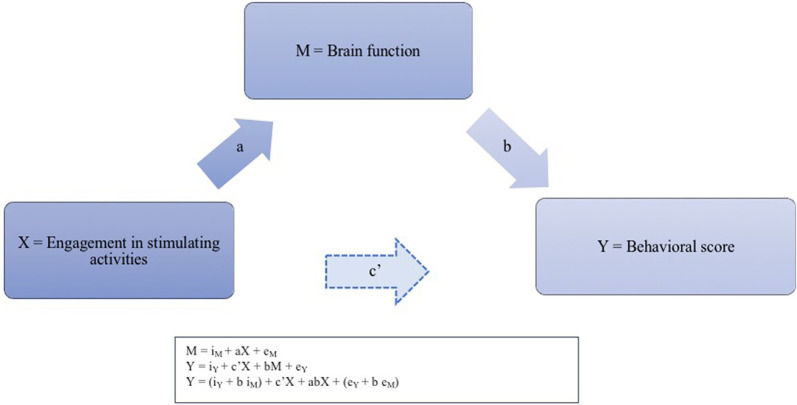
Illustration of the suspected neurofunctional implementation of the reserve phenomenon. For each pair of regions presenting an functional connectivity (FC) strength significantly associated with accuracy, we thus calculated the four pathways of the mediation model. Pathway (a) examined the regression coefficient for the effect of engagement activities on task-induced FC, pathway (b) determined the association between FC and accuracy, and pathway (c) and (c’), respectively, assessed the total (indirect) and direct effect of engagement activities on accuracy. The three successive regression equations for testing the mediation effect between the level of engagement in stimulating activities (X) and behavioral score (Y) through brain function (M) are presented in the caption: regressing the mediator on the independent variable; regressing the dependent variable on the independent variable, and regressing the dependent variable on both the independent variable and on the mediator.

By definition, aging does not occur overnight, and reserve probably builds slowly over the years (Fritsch et al., 2007; Schneider-Garces et al., 2010; Jefferson et al., 2011; Fabiani, 2012; Steffener and Stern, 2012; Cabeza et al., 2018). Lifelong engagement in stimulating activities may shape the brain circuitry, and older adults may rely on different brain circuitry than younger adults to perform a given task (Scarmeas and Stern, 2003; Habeck et al., 2015). The mechanistic expression of reserve in the adult brain is therefore expected to vary with age (Stern et al., 2008; Bastin et al., 2012; Fleck et al., 2017). From this perspective, more active engagement in stimulating activities would be expected to promote favorable structural and functional changes, increasing the brain’s ability to cope with the aging and in turn helping it to maintain cognitive performance. In other words, the hypothesis is that engagement in activities indirectly impacts cognitive performance by mediating the brain’s functional organization (Figure 1). However, this hypothesis still requires direct experimental validation. Studies exploring the neural implementation of reserve mostly point to increased neural efficiency during task performance and increased capacity when confronted with challenging tasks, but these studies selected tasks for which performance is known to decline with age, such as working memory, attention, or inhibition tasks (Stern, 2009; Steffener and Stern, 2012; Oh et al., 2018). In this way, previous research offered insights into interindividual differences in challenging situations but does not indicate how a cognitive ability is maintained across the adult lifespan.

The phenomenon of functional connectivity (FC) allows one to explore how different regions of the brain work together. Different regions of the brain do not function in isolation; rather, cost-efficient mechanisms for information transfer between distant regions are favored (Sporns, 2014). FC thus has the potential to reveal the neurofunctional mechanisms believed to indicate accumulated expertise and greater efficiency due to experience, such as increased segregation of brain regions (Marques et al., 2016; Lee et al., 2019). For example, default mode network (DMN) negative synchronization -or decoupling- from task-related regions is considered to be a key mediator of cognitive performance (see Sala-Llonch et al., 2015, for a review), and increased synchrony between brain regions related to semantic processing has been suggested to support cognitive performance in healthy aging (Wirth et al., 2011; Hoyau et al., 2018; Spreng and Turner, 2019). FC has also proven useful to highlight age-specific functional properties that support optimal performance (see Antonenko et al., 2013, for a review). Cognitive ability is the result of complex neurofunctional activity between regions that can be captured at different topographical and functional scales in various contexts. So far, age-related changes in FC have mainly been explored using a resting-state condition (e.g., looking at a fixation cross; Dennis and Thompson, 2014; Anthony and Lin, 2018). FC has proven sensitive to measures of the impact of engagement in stimulating activities, both in healthy aging (Bastin et al., 2012; Arenaza-Urquijo et al., 2013; Marques et al., 2015; Fleck et al., 2017; Perry et al., 2017; Lee et al., 2019) and in neurodegenerative pathology (Bozzali et al., 2015; Franzmeier et al., 2016, 2017; Lee et al., 2019). In older individuals, evidence suggests that one set of correlated regions is closely related to education, including visual, dorsolateral prefrontal, salience, somatomotor, and default and control networks (Perry et al., 2017).

In young adults too, a study of 461 individuals reported one strong component linking lifestyle, demographic and psychometric measures to each other and to FC in regions that, taken together, have a high degree of spatial overlap with the DMN (Smith et al., 2015). These studies show the potential relationship between changes in FC and the existence of conditions favoring a reserve, but they do not offer specific insight into the cognitive benefits of the reserve. There is now a consensus that it is important to explicitly link reserve measures and neurofunctional proxies to actual cognitive performance rather than a resting-state condition (Steffener and Stern, 2012; Cabeza et al., 2018).

There is strong evidence that the manifestation of the reserve may depend on the type of cognitively stimulating activity one is engaged in (Bosch et al., 2010; Steffener et al., 2014; Darby et al., 2017; Anthony and Lin, 2018; Spreng and Turner, 2019). Concerning cognitive task performance, the reserve has primarily been explored in cognitive domains that typically decline with age, such as memory (e.g., Anthony and Lin, 2018). However, other cognitive abilities are much less prone to decline with age, such as word knowledge and the ability to name objects (Verhaegen and Poncelet, 2013). Indeed, accuracy in vocabulary measures is generally maintained or even increased until at least 65 years of age (Salthouse, 2014), as the relationship between age and semantic knowledge degradation follows a quadratic function (Catricalà et al., 2015). Naming accuracy thus appears to be a long preserved ability despite an increase in response time (Wierenga et al., 2008) and a complaint regarding the retrieval of proper names (Condret-Santi et al., 2013), which may be associated with a general slowing down in information processing (Feyereisen et al., 1998). Yet, the naming of common names is not a frequent complaint in healthy aging or when impaired, it may be an early sign of a pathological process (Bowles et al., 1987; Jacobs et al., 1995). Exploring functional correlates underlying accuracy in a confrontation naming task may therefore constitute a suitable framework for the observation of a neurocognitive reserve phenomenon. Naming relies on an extended neurofunctional circuit, including regions of both dorsal and ventral pathways, which are in charge of speech production and semantic processes, respectively (Price, 2012; Duffau et al., 2014). Language networks appear more specific during an active task compared to the same connections during resting-state (Jackson et al., 2015; Tran et al., 2018). In this context, task-induced FC provides information specific to the cognitive task at hand and allows for better online monitoring of task performance (Campbell and Schacter, 2016; Geerligs et al., 2017), justifying its use in the study of the potential neurocognitive reserve phenomena subserving naming performance.

The study of task-induced FC in task-related regions represents an exciting candidate for the indirect measurement of the neurocognitive reserve. A mediation analysis uses an indirect effect model to test the hypothesis of a process through which one variable associate with another, using a combination of linear regression analysis. A mediation model thus allows us to determine the extent to which the association between reserve proxies and naming accuracy is dependent on task FC. In this view, proxy measures of reserve would indirectly support cognitive performance by their impact on brain functional organization, which mediates the indirect relationship between engagement in stimulating activities and cognitive performance (Figure 1). While this mediation model is based on empirical and theoretical evidence, to our knowledge, it has never been tested using task-induced FC to examine a cognitive ability that is generally preserved in aging. This kind of mechanistic demonstration would be a first step in revealing the relationships between reserve proxies, brain function, and preserved naming in aging.

The objective of this exploratory study is 2-fold: (1) to describe the task FC patterns that sustain preserved performance in a naming task across both ends of the adult life continuum; and (2) to determine whether these neurofunctional patterns account for the effects of neurocognitive reserve proxies on naming accuracy, using mediation analysis.

Given that more segregated FC involving the DMN has been shown to support cognitive performance (Lee et al., 2019), a decrease in the coupling between the DMN and the brain regions involved in the task is expected concerning naming accuracy, signaling efficient or economic processes during task performance. Alternatively, increased synchrony between task-related regions could be associated with accuracy, indicating greater reliance on specialized processes for the cognitive activity in question (Turner and Spreng, 2015). During naming, perceptual, phonological, and semantic processes can be expected to support task performance (Price, 2012).

An influence of reserve, expressed by an FC mediation effect, is expected between engagement in stimulating activities and accuracy. The effect may be expressed in both young and older individuals, given that there may be a continuum of cognitive benefit from lifestyle engagement throughout the life span (Cabeza et al., 2018). Conversely, the effect of the reserve during naming may be expressed differently as a function of age, suggesting that it is an age-specific phenomenon (Fleck et al., 2017).

## Materials and Methods

### Study Sample

A total of 72 potential participants gave their informed consent to participate in this study, following local ethics committee guidelines at the CRIUGM. Participants were native French-speakers, right-handed, and free of neurological disorders, a history of drug or alcohol dependency, major depression, or moderate to severe auditory or visual disorders. During recruitment, particular attention was paid to the number of years of education, to equally distribute the individuals who had completed at least a college education (13 years of education in Quebec) in each group and obtaining a sample that is representative of a large proportion of the population. In sum, the percentage of participants who had completed 13 years or fewer of formal education was 46.9% among the younger adults and 54.8% among the older adults. All participants underwent cognitive and hearing screening tests. The RAVEN 9-Item test (Bilker et al., 2012) was used to ensure equivalent nonverbal intelligence among our groups. For older adults, screening also included the Montreal Cognitive Assessment (MoCA; Nasreddine et al., 2005) to eliminate any candidates with MCI, with a standard cut-off score of 26 and a pure-tone test to ensure hearing acuity within ISO Standard 7029:2000. In sum, there were no age-related differences in cognitive and sociodemographic characteristics, except for age, sex, and occupation (Table 1). The final sample of candidates with complete data who passed the quality control test for fMRI preprocessing included 29 older adults and 35 younger adults.

**Table 1:** Cognitive and sociodemographic characteristics of the sample.

	Younger adults Mean* (SD)* [min-max]	Older adults Mean* (SD)* [min-max]
Sociodemographic characteristics		
Mean age (SD)*	26.41 *(5.22)* [18–35]	69.97 *(5.50)* [61–85]
Sex (male/female)*	20/15	12/17
Mean years of education *(SD)* [min-max]	14.09 *(3.26*) [9–20]	14.26 *(3.29)* [11–21]
% of participants < 13 years of formal education	46.9%	54.8%
Occupational complexity (ISCO-08 scale) *(SD)* [min-max]*	8.13 (3.26) [2–11]	3.55 (2.5) [1–11]
Engagement in stimulating activities index *(SD)* [min-max]	−0.05 (0.56) [−1.27–1.08]	−0.03 (0.59) [−1.23–1.12]
Behavioral performance *(SD)* [min-max]		
BNT accuracy *(SD)* [min-max]	44.67 *(7.69)* [24–57]	44.55 *(5.55)* [34–55]
RAVEN 9-Item accuracy *(SD)* [min-max]	3.97 *(2.00)* [1–8]	4.16 (*2.08*) [1–8]

*between-group significant differences in cognitive and sociodemographic characteristics using independent samples *T*-tests (*p* < 0.05).

### Neurocognitive Reserve Proxies and Cognitive Assessment

#### Engagement in Stimulating Activities Index (EAI)

Multiple variables have been used throughout the literature (for a review see Valenzuela and Sachdev, 2006; Grotz et al., 2017), but those which have been the subject of the majority of studies have been education level, professional level and one’s level of participation in cognitively stimulating activities. Such markers are not in any way direct measures of the reserve or its mechanisms and remain very gross estimates. Consequently, several studies suggest using such markers in combination rather than in isolation (Siedlecki et al., 2009; Jones et al., 2011; Consentino and Stern, 2013; Grotz et al., 2017). A compound indicator may provide a more precise measure of reserve than individual measures, limit potential collinearity issues, and partially offset the fact that individual proxy measures may not be fully orthogonal (Siedlecki et al., 2009; Jones et al., 2011; Opdebeeck et al., 2015). The engagement in activities index (EAI) was thus defined as the averaged *z*-score for three values: years of education, the total score on the Victoria Longitudinal Scale (VLS) Activity Lifestyle Questionnaire, and inverted occupation ranking.

Years of completed formal education was used as a widely accepted proxy for cognitive reserve. Occupation complexity was measured by the ISCO-08 scale (International Labour Organization, 2008). The ISCO-08 classifies occupations based on a 12-point scale. Lower scores indicate higher occupational complexity (e.g., legislators and senior officials), and thus indicate greater cognitive stimulation in the workplace. Consequently, values for this scale were inverted. The VLS Activity Lifestyle Questionnaire is a 64-item questionnaire developed and validated to obtain a multifactorial measure of the various activities that may support healthy lifestyles (Hultsch et al., 1999), such as physical activities, self-maintenance, social activities, hobbies, passive information-processing, and active information-processing activities. Participants rated their typical frequency of participation over the last year on a nine-point scale (0 denoting no activity). Items were summed to produce a composite activity measure.

#### Behavioral Analysis

The Boston Naming Test (BNT; Goodglass et al., 1983) is among the most widely used visual confrontation naming tasks in both clinical and experimental settings. Moreover, performance on this test is not significantly altered by age (LaBarge et al., 1986). Picture naming, therefore, provides a basis for developing knowledge of the neural constituents of preserved naming abilities in aging. Accuracy (number of correct responses given in the maximum time window) in all naming trials was analyzed. Three trained raters independently scored participants’ accuracy according to validated norms (Roberts and Doucet, 2011) and reached a consensus. SPSS was used for statistical analysis (IBM SPSS Statistics for Macintosh, Version 25.0, 2017. Armonk, NY, USA: IBM Corp.).

To initially confirm that older adults actually performed successfully on naming, the two age groups’ total scores were compared using independent sample *t*-tests: similar accuracy scores were observed for younger (*M* = 44.67, SD = 7.69) and older (*M* = 44.55, SD = 5.55) adults, *t*_(64)_ = 0.068, *p* = 0.95. Table 1 presents the behavioral results.

### fMRI Analysis

#### Task Design

Participants were asked to complete the overt object naming task (BNT) during fMRI data acquisition. They were required to name the pictures they saw on the screen aloud as fast as possible. An overt answer was preferred over a covert one, to better control for the correctness and reflect the actual cognitive processes at play during acquisition, including speech output and sensory feedback (Campbell and Schacter, 2016).

A total of 145 volumes were acquired while participants performed the task in the scanner. Each of the 60 BNT stimuli was presented for 1,500 ms and participants had another 1,500 ms to answer before the next trial. Pilot acquisitions with both young and healthy adults were acquired before settling on the final task paradigm. A long response time duration was preferred as it is only after 700 ms that neurofunctional response to the tip-of-the-tongue phenomenon is typically observed (Shafto and Tyler, 2014) and as suggested by the pilot acquisitions. An interstimulus interval of 350 ms separated the stimuli. A fixation cross indicated the end of the trial. The task was composed of 12 blocks, each of which lasted 17.5 s and contained five images to name. The blocks were separated by rest epochs (fixation cross) lasting 17.5 s.

#### MRI Scanning and Data Processing

MRI images were acquired with a 32-channel head coil and a 3T SIEMENS TrioTim magnetic resonance imaging system. Participants were instructed to limit their movements as much as possible, and a practice session was carried out before the scanning session. Foam rubber pads within the head coil also restricted head movement. Earplugs were provided to dampen the noise conditions for the participants in the scanner. A microphone was oriented toward the participant’s mouth to allow vocal recording (MRConfon^TM^). When necessary, participants’ vision was corrected using MRI-175 compatible lenses that matched each’s prescription. The task stimuli were presented using DMDX presentation software (Forster and Forster, 2003).

Anatomical images (T1) were acquired with a Multi Echo Multi Planar Rapid Gradient Echo (MEMPRAGE) pulse sequence and a GRAPPA (GeneRalized Autocalibrating Partial Parallel Acquisition) acceleration factor using the following parameters: FoV = 256.0 mm^2^, matrix size = 256 × 256, 176 slices covering the whole brain, 1 mm isotropic voxel size, TE/TR = 1.64/253 ms, flip angle = 7.0°.

Functional images (T2*) were acquired with echo-planar imaging (EPI) pulse sequence and a GRAPPA acceleration factor using the following parameters: FoV = 220 × 220 mm, matrix size = 74 × 74, 50 ascending slices covering the whole brain, 3 mm isotropic voxel size, TR/TE = 3,000/20 ms, flip angle = 90°. T2* image acquisition was oriented to –30° from the AC-PC line (to reduce signal loss from the anterior temporal lobes). The first five volumes of each run were automatically discarded during acquisition.

#### Preprocessing

Structural and BOLD functional metrics were preprocessed using SPM12^1^ and CONN toolboxes (v.18.b^2^) implemented in MATLAB 2015b^3^. Functional images were coregistered and realigned using the CONN preprocessing pipeline. Coregistered structural (T1) images were segmented into gray matter, white matter, and cerebrospinal fluid with a sampling of 1.5 × 1.5 × 1.5 mm using trilinear interpolation as implemented in the VBM12 toolbox in SPM12^4^. Total gray matter volume (TGMV) was extracted at this step to be used in further analysis steps. Images were normalized using Diffeomorphic Anatomical Registration Through Exponentiated Lie (DARTEL; Ashburner, 2007) to create a custom template. All realigned images and the co-registered structural images were warped into the Montreal Neurological Institute (MNI) space using the flow field images obtained with the DARTEL template. Images were modulated by multiplying the Jacobian deformation parameters defined during normalization to preserve the total amount of original gray matter before normalization (Ashburner and Friston, 2005; Zhu et al., 2014). Smoothing with a 6-mm full-width half-maximum (FWHM) isotropic Gaussian kernel was applied on the warped images, which were then normalized in the MNI space. Functional images were coregistered to the structural T1 images. Smoothed normalized images were entered in the CONN toolbox.

The ART-based functional outlier detection option (motion censoring procedure) was implemented to remove unwanted motion, physiological, and other artifactual effects from the BOLD signal (Mazaika et al., 2005). The scrubbing threshold was established as 0.9 mm scan-to-scan head motion or global signal intensity >5SD above the mean signal for the session (Mazaika et al., 2009; Whitfield-Gabrieli and Nieto-Castanon, 2012). A dummy variable represented each outlier in the first level denoising step. Three participants who presented more than 25% of outliers in their total volumes were excluded from further FC analysis. An anatomical-component-based noise correction method (aCompCor) was applied (Behzadi et al., 2007), regressing the white matter and cerebrospinal fluid from the BOLD signal. The six realignment parameters (with their first temporal derivatives) and the task effect (BOLD time series orthogonalization to task effects) were added as regressors in the model. A high pass filter (<0.01 Hz) was then applied (Satterthwaite et al., 2013; Muschelli et al., 2014).

Quality assessment was performed before and after denoising, by visually inspecting the overlay of functional and structural individual realigned images, the overlay of the functional images and the MNI template, as well as the BOLD functional time points movie and distribution of scrubbed volumes across time. One participant was excluded from further FC analysis because of an isolated but massive movement. No other particularities were noted.

In total, four participants were excluded from the initial 72. The final sample was composed of 68 individuals: 37 young adults and 31 older adults (Table 1).

#### Functional Connectivity Analysis

The CONN toolbox was used to compute seed to voxel FC analysis independently in younger and older adults. FC values for the pairs of regions presenting a main effect of accuracy were extracted and used later in the mediation analysis step.

#### ROI Definition

Regions of interest (ROIs) were selected for seed-based connectivity to reduce the number of observations and guide the FC data analysis.

The atlas provided by the CONN toolbox is composed of functionally coherent regions derived from an independent component analysis of the Human Connectome Project (HCP) data set of 497 subjects (Whitfield-Gabrieli and Nieto-Castanon, 2012). Publicly available ROIs enhance comparability with other groups and minimize the bias of manually selecting components from our small sample. On the other hand, previous work demonstrated that sample- and task-specific parcellation was more functionally relevant (Dickie et al., 2017; Geerligs et al., 2017; Salehi et al., 2017, 2018). To achieve a balance between sensitivity and specificity, a three-step methodology was used, combining a sample analysis of the task main effect along with spatial and time mapping with CONN parcellation.

**Figure 2:**
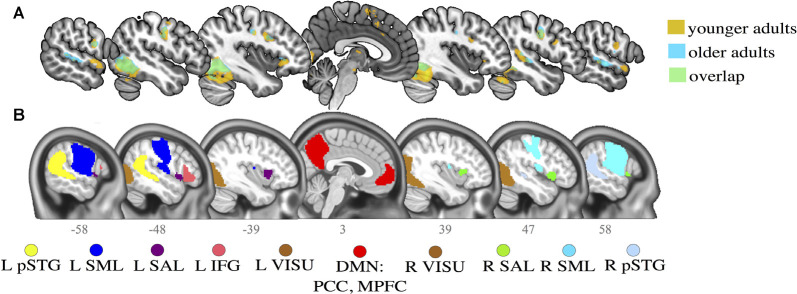
Activation maps for younger and older adults and selected regions of interest (ROI) seeds. **(A)** Activation clusters in the main contrast of interest (task > rest) for both younger (in orange) and older (in blue) adults. Overlaps (in green) are generally observed between younger and older adults in the task contrast. **(B)** Networks defined by CONN toolbox. Slice numbers are indicated on the sagittal axis. pSTG, posterior superior temporal gyrus; IFG, inferior frontal gyrus; SML, lateral sensorimotor network; VISU, lateral visual network; SAL, salience anterior insula; PCC, posterior cingulate cortex; MPFC, middle prefrontal cortex. L, left; R, right.

First, voxel-wise T-maps were constructed for each subject using a task > rest contrast (task main effect). Second-level group analyses were also performed to test for significant differences across groups. None were found except in the right inferior cerebellum (MNI: 27-54-46), but this cluster was included in a more massive occipital cluster in the union activation maps and thus was not selected as a distinct seed in FC analysis. Activation peaks of the union of the task main effect maps (*t*-tests) for both age groups were extracted. Finally, these activation peaks were overlapped with the CONN networks in the MNI space. Networks that intersected with one or more task activation peaks were selected as seeds for further FC analysis (Figure 2).

An additional validation step was completed, to ensure functional coherence within the groupings: a one-sample *t*-test was computed for all participants during the task condition (ignoring rest epochs) to identify average connectivity patterns of all activation peaks and CONN networks. Then, an ROI-to-ROI hierarchical clustering algorithm sorted all regions to identify ROIs with similar time series, using an FDR connection-level threshold of *p* < 0.0001 at the analysis level (Supplementary Figure 1).

While language abilities likely engage a large number of brain areas, some regions respond more specifically and strongly when performing word production tasks. The selected regions figure among the canonical language network (Price, 2012; Tomasi and Volkow, 2012; Tran et al., 2018): the right and left posterior superior temporal gyrus (R and LpSTG), the left inferior frontal gyrus (LIFG); the lateral sensorimotor network (R and LSM); the lateral visual network (R and LVISU); and the salience network (L and R anterior insula).

Two additional ROIs were selected in key regions of the DMN that consistently show an association with age or cognitive performance (Andrews-Hanna et al., 2007; Damoiseaux et al., 2008; Geerligs et al., 2014; Ng et al., 2016; Chan et al., 2017; Kong et al., 2019): the middle prefrontal cortex (MPFC) and posterior cingulate cortex (PCC). Peak coordinates for the task contrast and the associated CONN networks are provided in Table 2.

**Table 2:** Seed definition.

Anatomical region	MNI coordinates of peak activation (mm)			Cluster size	T peak value	CONN overlapping network
	x	y	z			
Left occipital	−40	−72	−12	7118	15.38	LVISU
Right occipital	27	−80	−10	6,920	16.48	RVISU
Left posterior superior temporal gyrus	−60	−39	6	65	9.41	LpSTG
Right posterior superior temporal gyrus	50	−34	8	70	9.41	RpSTG
Right middle temporal gyrus	54	−24	−2	126	10.76	RpSTG
Left opercular inferior frontal gyrus	−38	6	30	94	10.19	LSAL
Left orbital inferior frontal gyrus	−30	26	2	336	11.86	LIFG
Right orbital inferior frontal gyrus	33	27	−2	81	11.98	RSAL
Right insula	34	18	8	49	10.72	RSAL
Left primary motor area	−44	−12	36	249	11.39	LSML
Right primary motor area	50	−4	39	242	11.13	RSML
Left somatosensory cortex	−22	−66	36	159	10.48	LSML
Left premotor cortex	−60	2	21	133	10.83	RSML

#### Task-Induced Functional Connectivity Analysis and Association with Performance

The spatial topography of seed-to-voxel FC was then examined in an HRF-weighted generalized linear model (controlling for hemodynamic response function, or HRF) using the 10 networks identified by previous steps as seeds. Correlation coefficients between each seed time series and all other voxels were calculated and transformed into Fischer’s *Z*-scores to increase normality. Regressions of naming accuracy scores onto FC during the task condition (ignoring the rest epochs) were then carried out in each group separately, with age, gender and TGMV entered as nuisance variables. Correction for multiple comparisons was done using a combined voxel-level height threshold (*p* < 0.001 uncorrected) and a cluster extent threshold (*p* < 0.05 FWE corrected). The FC strength (Fischer transformed correlation coefficient) was then extracted from each pair of regions presenting a significant association with performance in each group separately to further test the mediation model. Automatic labels were provided by CONN using the anatomy toolbox v2.0 (Eickhoff et al., 2015) and further manual verification was implemented with the AAL, Tzourio-Mazoyer, and Brodmann atlases.

### Mediation Analysis

For each pair of regions that FC significantly associated with performance, a mediation pathway analysis (Hayes, 2017) was implemented using the PROCESS macro in SPSS (v.3.4^5^) to explore the potential mechanistic effect of engagement in cognitive activities on task accuracy through FC. The mediation analysis method is illustrated in Figure 1.

According to the method described by Baron and Kenny (1986) and Hayes (2017), three regression equations need to be tested to establish a mediation effect: regressing the mediator on the independent variable; regressing the dependent variable on the independent variable, and regressing the dependent variable on both the independent variable and on the mediator. For each pair of regions presenting an FC strength significantly associated with accuracy, we thus calculated the four pathways of the mediation model. Pathway (a) examined the regression coefficient for the effect of engagement activities on task-induced FC, pathway (b) determined the association between FC and accuracy, and pathway (c) and (c’), respectively, assessed the total (indirect) and direct effect of engagement activities on accuracy. Each path represents a causal relationship between two variables, to which a coefficient—the standardized regression coefficient or beta—is calculated. To demonstrate a mediation effect, the following conditions must be met: (1) the independent variable must associate with the mediator in the first equation; (2) the independent variable should associate with the dependent variable in the second equation; and (3) the mediator must associate with the dependent variable in the third regression.

PROCESS relies on a bias-corrected estimation of the mediation effect, using a bootstrapping method with 5,000 samples and correcting for asymmetry in the distribution of scores (Hayes, 2017). Significance was established when 95% of bias-corrected and accelerated bootstrap confidence intervals (BCa CI) excluded zero (Preacher and Hayes, 2008). The standardized regression coefficient of the indirect effect was computed to obtain an estimate of its effect size. To ascertain that the presence or absence of an indirect effect was not the result of choosing a composite index of engagement in cognitive activities instead of a single metric, analyses were repeated using years of education alone.

## Results

### Association Between Naming Accuracy and Functional Connectivity in Older Adults

Regression models including FC metrics and accuracy and response time were computed for each ROI and each age group.

**Figure 3:**
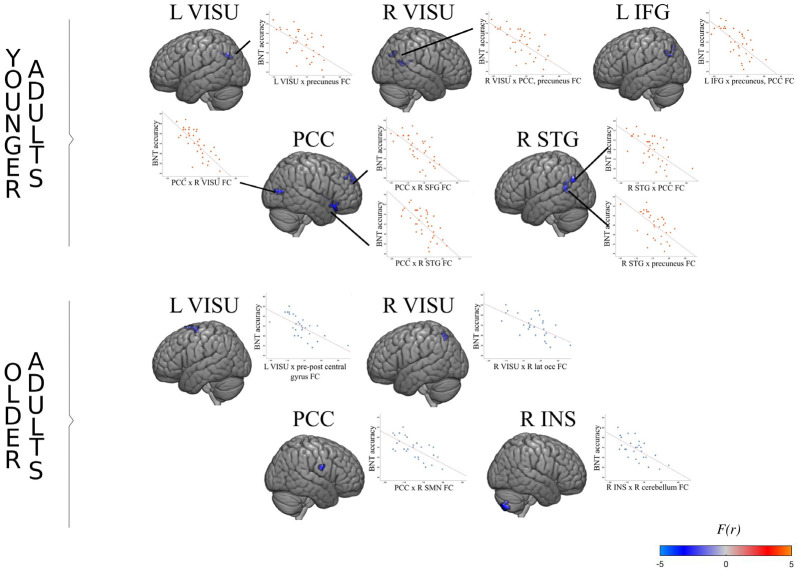
FC association with performance in younger and older adults. For each age group, the surface maps show brain regions which degree of connectivity with the seed region (indicated on top) varies as a function of naming accuracy during the task. The scatterplot shows the average regression slope between accuracy (total number of good answers) and connectivity (the correlation coefficient between two regions). For example, the left visual seed exhibits a significant negative association with a cluster located in the posterior default mode network (DMN) in younger adults. In other words, younger adults who perform better also show decreased synchronization between the left visual cortex and the posterior DMN.

As expected, the connectivity of the posterior DMN (PCC and precuneus) was inversely associated with naming accuracy in both age groups during the task: the lower the connectivity, the more the performance. However, the effect involves different regions in older adults and younger adults, as illustrated in Figure 3. Older adults with better accuracy scores tended to show a functional decoupling between regions of the posterior DMN and right superior motor areas (pre-post central gyrus), the right lateral sensorimotor network, and the right lateral occipital visual network. Accuracy is also associated with coupling decreases between the left lateral occipital visual network and bilateral frontal motor areas, as well as between the right anterior insula and the right cerebellum. Younger adults, on the other hand, relied on the posterior DMN decoupling from the bilateral superior frontal gyrus, right superior temporal gyrus, and bilateral visual network to mediate accuracy.

### Indirect Mediation Effect of Engagement in Cognitive Activities on Naming Accuracy Through FC

The mediation of engagement in cognitive activities on naming accuracy through FC was significant only in younger adults. PCC decoupling strength significantly mediated the effect of engagement activities on performance associated with the bilateral frontal orbital and left IFG pars triangularis [ab = 0.19, BCa CI (0.02 0.35)], the right orbitofrontal pole and insula [ab = 0.22, BCa CI (0.04 0.39)], and the right lateral occipital cortex [ab = 0.22, BCa CI (0.03 0.43)].

**Figure 4:**
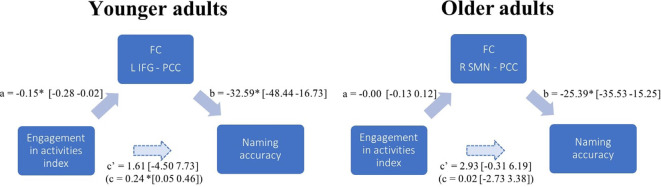
FC mediation effect in younger and older adults. Path diagram illustrating the results from one mediation model in each age group. The examples show how PCC connectivity to the L IFG mediates the association between the level of engagement in stimulating activities (EAI) and naming accuracy in younger adults; while there is no such significant mediating effect of PCC connectivity to the R SMN in older adults. Each arrow in a path diagram represents a causal relationship between two variables to which a coefficient (the standardized β-weights derived from linear regression) is assigned. Confidence intervals are indicated in brackets. Significance was established when 95% confidence intervals excluded zero (Preacher and Hayes, 2008). For each pair of regions presenting an FC strength significantly associated with accuracy, we calculated the four pathways of the mediation model. Pathway examined the regression coefficient for the effect of EAI on task-induced FC, pathway b determined the association between FC and accuracy, and pathway c and c’, respectively, assessed the total (indirect) and direct effect of engagement in activities on accuracy. In the example given for the younger adults’ mediation model (PCC-L IFG), there is no direct association between the engagement in stimulating activities and naming accuracy c’ path, a significant negative association in the a and b paths, and a significant total indirect effect (c path). In the older adults’ mediation model, contrary to younger adults, the association between EAI and task FC (a path) and the total indirect effect (c path) were non-significant.

Engagement in cognitive activities also had a significant effect on naming accuracy through FC of the language network, LIFG and PCC [ab = 0.24, BCa CI (0.16 1.28)]; the pSTG and PCC-precuneus [ab = 0.24, BCa CI (0.04 0.45)] and left superior lateral occipital cortex [ab = 0.30, BCa CI (0.11 0.46)]; the left visual network and precuneus [ab = 0.24, BCa CI (0.08 0.41)]; and the right visual network and precuneus-PCC [ab = 0.35, BCa CI (0.17 0.55)]. One significant mediation effect is illustrated in Figure 4 as an example. Other effects were similar, with no direct association between the EAI and naming accuracy, a significant negative association in the a and b paths, and a significant indirect effect.

The analyses were repeated using years of education alone instead of the EAI, and the results remained similar in both groups.

## Discussion

The main goal of this study was to explore the potential role of FC in mediating the indirect effect of experiences acquired throughout the life span—such as education, occupation, and lifestyle—on naming accuracy. The results demonstrate, first, a clear association between functional decoupling of the posterior DMN and naming accuracy during task performance in both young and older adults. An FC pattern specific to older adults revealed that high-performing older adults rely more on the segregation of the PCC and motor control regions. In turn, in younger adults with greater accuracy, the activity of the PCC is decoupled from the frontal, occipital and temporal areas, which are key areas in a naming task. The main result shows that, in younger individuals but not in older ones, the FC pattern supporting cognitive performance is also associated with quantitatively richer engagement in cognitive activities. The cognitive benefit of engaging in stimulating activities and the consequent shaping of the neurofunctional organization is therefore demonstrated in younger but not older adults.

### Functional Connectivity and Task Performance

FC analyses confirmed the initial expectation that the posterior DMN (i.e., PCC and precuneus) plays a dynamic role during task execution in both younger and older adults, supporting performance using negative synchronization with other brain regions. The involvement of PCC decoupling in task performance has a long history (Andrews-Hanna et al., 2007; Persson et al., 2007; Sambataro et al., 2010; Campbell et al., 2013; La et al., 2016; Vatansever et al., 2017). The PCC is believed to be involved in the retrieval of stored knowledge, and its decoupling might generally favor engagement in an externally focused task (Buckner et al., 2008). The precuneus is another core area of the posterior DMN (Fransson and Marrelec, 2008; Cole et al., 2010), and its functional segregation was previously demonstrated to correlate with memory scores, even after correcting for the effect of age (Sala-Llonch et al., 2015).

However, there are also striking differences in the segregation patterns of each age group. In association with accuracy, the PCC-precuneus activity of younger adults decouples from a broad set of regions typically involved in controlled semantic processing and word retrievals, such as the inferior frontal and superior temporal regions (Price, 2012).

In contrast, in less performing older adults, there is an increased correlation between the PCC and speech motor regions (i.e., lower-performing older adults show a positive correlation while higher-performing older adults show an anticorrelation), as well as between the visual cortex and frontal motor areas, and the insula-cerebellum. The left visual occipital cortex and inferior frontal areas represent the classic picture-naming pathway, responsible for picture recognition and voluntary motor movements, respectively (Duffau et al., 2014). The insula and cerebellum are respectively involved in semantic processing and error detection in speech production; together, they act at the interface between phonological and semantic processes (Ardila et al., 2014; Riedel et al., 2015; Fiez, 2016). Impairments of both the insula and cerebellum can lead to the tip-of-the-tongue phenomenon and phonological errors (Bernard and Seidler, 2014; Shafto and Tyler, 2014).

It may be hypothesized that older adults, compared to younger adults, rely less on PCC segregation because their accumulated semantic knowledge allows them to process the semantic aspects of the task more automatically. Indeed, the PCC is well known for its active role in accessing prior verbal knowledge (Yonelinas et al., 2005; Binder et al., 2009; Price, 2012; Basso et al., 2013). It has previously been demonstrated that, when access to semantic knowledge is relevant to the task, there is greater default-executive coupling in older than in younger adults (Spreng et al., 2014; Adnan et al., 2019). The fact that older adults rely on less widespread decoupling from language-related regions than younger adults may therefore reflect their greater reliance on PCC-language network connectivity to access semantic knowledge. Meanwhile, given older adults’ generally slower response times during naming (Thomas et al., 1977; Feyereisen et al., 1998), the critical decoupling between premotor regions and the PCC may well signal a motor control challenge, resulting in a strong reliance on speech-motor control regions to achieve naming performance in older adults (Tremblay et al., 2017). Further investigation of a wide variety of cognitive domains and task demands will be required to understand task-specific FC mechanisms.

### Mediation of Engagement in Cognitive Activities on Cognitive Performance Through Functional Connectivity

An indirect effect of the reserve on accuracy was expected in both young and older individuals, assuming that the beneficial effect of engagement in stimulating activities represents a continuum. However, in this study, an association between engagement in cognitive activities and cognition was observed only in younger adults. The expression of the reserve, as measured by FC during task performance, therefore appears to be age-specific.

There are numerous potential explanations of the observation of reserve-related mechanisms through FC in younger adults alone. This is not the first time that such a finding has been reported. Stern et al. (2008) also found a significant association with reserve only in young individuals, which led them to suggest that there is a general neural instantiation of reserve in younger adults, irrespective of performance or task demand: a latent neural resource available for later use in challenging situations. In older age, reserve expression may indeed become dependent on the actual demand of the task, modulated by the brain’s capacity to cope with task demand (Stern et al., 2008; Reuter-Lorenz and Park, 2014; Cabeza et al., 2018). The impact of demand level is evidenced in studies that compare healthy individuals with individuals presenting a clinical condition. A recent review noted that reserve-related compensatory mechanisms were more commonly observed in MCI and Alzheimer’s disease than in healthy aging (Anthony and Lin, 2018). In another study, PCC decoupling was modulated by education in Alzheimer’s disease patients, less so in patients with MCI, and not at all in healthy subjects (Bozzali et al., 2015). In the context of a preserved naming ability, as observed in our sample of older adults, it might be the case that older adults do not need to exploit their reserve. In this view, the fact that the posterior DMN shows less critical decoupling concerning task accuracy in older than in younger adults may reflect an increased reliance on the posterior DMN to access previously stored knowledge. Older adults are known to benefit from previously accumulated semantic knowledge in a wide variety of tasks, relying on quasi-automatic processes (Spreng and Turner, 2019). The assessment of this hypothesis will require further comparison of the manifestation of the reserve with different cognitive contexts and degrees of complexity.

An alternative explanation might be that reserve—as measured by classical proxies—builds mainly in early adulthood and a saturation point concerning the benefits of cognitive engagement is reached at some point in the course of aging. For example, early education has been shown to contribute greatly to cognitive performance in later life by facilitating the brain’s ability to form segregated functional groups (Jefferson et al., 2011; Marques et al., 2016). Meanwhile, longitudinal findings show that high occupational complexity cannot compensate for the effects of low early educational attainment (Dekhtyar et al., 2015; Thow et al., 2017). In this view, the neurofunctional benefit of engaging in stimulating activities is more likely to be observable in younger adults, and the plateau effect may only be observed in middle age. Testing this hypothesis will require a lifelong perspective, with studies considering age as a continuous variable and using longitudinal designs to disentangle the complex relationships linking the reserve phenomenon to accuracy along the healthy adulthood continuum and in neurodegenerative disease.

From a methodological point of view, this exploratory study employs mediation analysis intending to unravel possible indirect relationships between life experience and cognitive performance, as was suggested by theoretical accounts of the neurocognitive reserve (Cabeza et al., 2018). To our knowledge, this proposition was never formally tested using cognitive abilities that remain well preserved with age. Such a pilot study yet present clear methodological limitations and will require further replication to validate the findings. Notably, the successive tests used for mediation analysis increase the risk of type I errors and the 5% significance threshold applied for each outcome does not allow for a complete correction for multiple testing. Alternatively, the relatively small sample size, combined with the important behavioral (Lindenberger and von Oertzen, 2006) and neurofunctional (Eavani et al., 2018) variability that characterizes aging, may have reduced the probability of significant discoveries. More generally, univariate analyses do not account for non-linear trends, as may be found in older adults (Au et al., 1995; Salthouse, 2014; Catricalà et al., 2015).

In our study, the use of a composite index was intended to account for numerous classical proxies of the reserve, and the use of a single proxy did not change the general outcome. Yet, the various proxy measures for engagement in cognitive activities may differ in their association with a cognitive function depending on the domain assessed (Jones et al., 2011; Opdebeeck et al., 2015). It may be that the proxy measures for reserve used in this study cannot identify exactly which lifelong experiences support older adults’ accuracy in a naming task. For example, Hoyau et al. (2018) recently found an impact of social leisure activities—and not individual activities—on naming response time, an effect partially mediated by brain activity. The respective contributions and critical levels for various proxy measures of the reserve will require further assessment concerning performance in different cognitive domains.

## Conclusion

This study, based on the most recent knowledge on the phenomenon of cognitive reserve, provided initial exploratory data regarding the indirect effect of engagement in cognitive activities on task accuracy through task-induced FC. While decoupling of the PCC was found to be crucial to task accuracy in both young and older adults, the indirect impact of engagement activities was only demonstrated in the younger group. This exploratory study, therefore, suggests that the beneficial impact of engagement activities on task-induced FC is evident at the younger end of the adulthood continuum, whereas the lifelong engagement of older adults in cognitive activities may reach saturation at some point in the course of aging.

## Data Availability Statement

The datasets generated for this study will not be made publicly available. Ethical approval did not include the authorization to publicly share the data or constitute a public database.

## Ethics Statement

The studies involving human participants were reviewed and approved by Comité Mixte d’Éthique et de la Recherche du Regroupement Neuroimagerie du Québec (CMER-RNQ). The patients/participants provided their written informed consent to participate in this study.

## Author Contributions

PF contributed to the conceptual and methodological development, the recruitment of participants, the acquisition of behavioral and neuroimaging data, the development of data analysis tools, the preparation, and analysis of behavioral and fMRI data, the interpretation of the results, and the drafting of the article. JJ contributed to the recruitment of participants, data preparation, and analysis in fMRI. SB contributed to the conceptual and methodological development, project funding, the interpretation of results, and manuscript revision. PB contributed to the conceptual development and revision of the manuscript (Ph.D. co-supervisor). YJ contributed to the conceptual development and revision of the manuscript, project funding (main Ph.D. supervisor). All authors contributed to the article and approved the submitted version.

## Conflict of Interest

The authors declare that the research was conducted in the absence of any commercial or financial relationships that could be construed as a potential conflict of interest.

## Footnotes

^1^http://www.fil.ion.ucl.ac.uk/spm/

^2^www.nitrc.org/projects/conn

^3^https://www.mathworks.com/

^4^http://dbm.neuro.uni-jena.de/wordpress/vbm/

^5^www.processmacro.org

## SUPPLEMENTARY MATERIAL

SUPPLEMENTARY FIGURE 1 Functional validation for the definition of the ROIs. ROIs 1–13 is the peaks of activation extracted from group T-maps of the main task effect. The fully named networks are provided by the CONN toolbox. The hierarchical graph groups time-series that show a similar pattern of functional connectivity. For example, as expected, activity in ROI 1 (R visual occipital lobe) is very strongly correlated with the CONN “Right Visual Lateral” network, less with the “Left Visual Lateral” network and not sign with the “Sensorimotor Lateral Network, ” suggesting the existence of a bilateral visual subnetwork.
