# Supplementary figures and images for: Functional Connectivity of Successful Picture-Naming: Age-Specific Organization and the Effect of Engaging in Stimulating Activities
Source: Front Aging Neurosci. 2020 Nov 5;12:535770. doi: 10.3389/fnagi.2020.535770 (PMC7674930; doi:10.3389/fnagi.2020.535770)

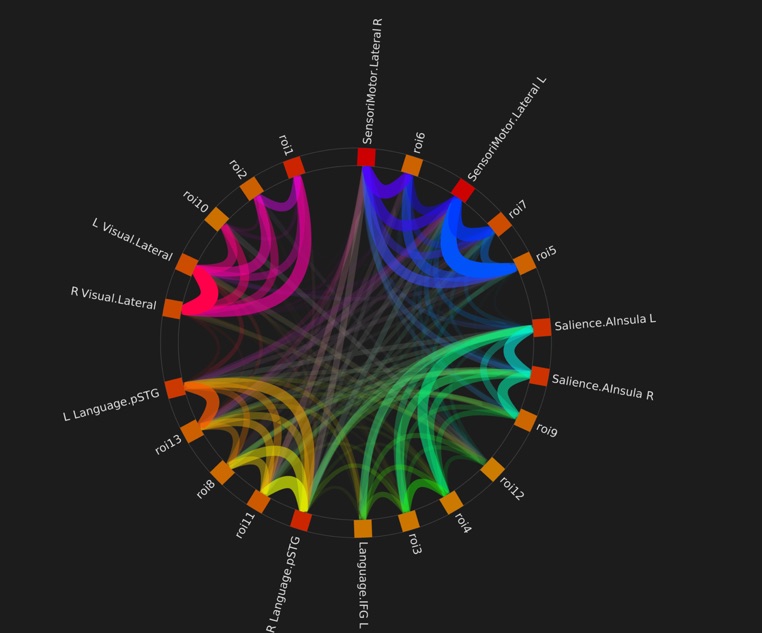

Supplement: SUPPLEMENTARY FIGURE 1 — Functional validation for the definition of the ROIs. ROIs 1–13 is the peaks of activation extracted from group T-maps of the main task effect. The fully named networks are provided by the CONN toolbox. The hierarchical graph groups time-series that show a similar pattern of functional connectivity. For example, as expected, activity in ROI 1 (R visual occipital lobe) is very strongly correlated with the CONN “Right Visual Lateral” network, less with the “Left Visual Lateral” network and not sign with the “Sensorimotor Lateral Network, ” suggesting the existence of a bilateral visual subnetwork. [file Image_1.JPEG]
